# Safety, Efficacy and Pharcacokinetics of Targeted Therapy with The Liposomal RNA Interference Therapeutic Atu027 Combined with Gemcitabine in Patients with Pancreatic Adenocarcinoma. A Randomized Phase Ib/IIa Study

**DOI:** 10.3390/cancers12113130

**Published:** 2020-10-26

**Authors:** Beate Schultheis, Dirk Strumberg, Jan Kuhlmann, Martin Wolf, Karin Link, Thomas Seufferlein, Joerg Kaufmann, Mathilde Feist, Frank Gebhardt, Mike Khan, Sebastian Stintzing, Uwe Pelzer

**Affiliations:** 1 Department of Hematology and Oncology, Marien Hospital Herne, University of Bochum, 44627 Herne, Germany; beate.schultheis@elisabethgruppe.de (B.S.); dirk.strumberg@elisabethgruppe.de (D.S.); 2 Department of Medicine II, University Hospital Freiburg, 79106 Freiburg, Germany; jan.kuhlmann@uniklinik-freiburg.de; 3 Department of Medicine, Hospital Kassel, 34125 Kassel, Germany; onkologie@klinikum-kassel.de; 4 Department of Medicine V, Hospital Nuernberg Nord, 90419 Nuernberg, Germany; Karin.link@klinikum-nuernberg.de; 5 Department of Medicine I, Universitätsklinikum Ulm, 89081 Ulm, Germany; thomas.seufferlein@uniklinik-ulm.de; 6 Silence Therapeutics GmbH, 13125 Berlin, Germany; J.Kaufmann@silence-therapeutics.com (J.K.); F.Gebhardt@silence-therapeutics.com (F.G.); 7 Department of Surgery, Charité-Universitätsmedizin Berlin, 13353 Berlin, Germany; mathilde.feist@charite.de; 8 Medical faculty, Humboldt-Universität zu Berlin, 10099 Berlin, Germany; 9 Berlin Institute of Health, 10178 Berlin, Germany; 10 Department of Biological Sciences, University of Warwick, Coventry CV4 7AL, UK; Michael.khan@warwick.ac.uk; 11 Department of Hematology, Oncology and Tumor Immunology, Charité-Universitätsmedizin Berlin, 10117 Berlin, Germany; sebastian.stintzing@charite.de

**Keywords:** pancreatic cancer, targeted therapy, RNA interfering, randomized trial

## Abstract

**Simple Summary:**

Pancreatic cancer is still a challenging disease, as chemotherapeutic options are limited. We present the data of a new liposomally formulated short interfering RNA, Atu027, which has antimetastatic activity. In this randomized trial, pharmacokinetics and efficacy were assessed in two different treatment schedules. Both types of treatment were well-tolerated, with the side effects mainly being laboratory abnormalities without clinical importance. The twice-weekly regime compared patients with locally advanced to those with metastatic disease; in the latter group, a significant prolongation of survival without disease progression could be found. Therefore, as Atu027 influences the vascular endothelium, we suggest the further investigation of this compound as it seems to be able to prevent or delay metastases of pancreatic cancer.

**Abstract:**

Background: Atu027 is a liposomally formulated short interfering RNA with anti-metastatic activity, which silences the expression of protein kinase N3 (PKN3) in the vascular endothelium. This trial was designed to assess the safety, pharmacokinetics and efficacy of Atu027 in combination with gemcitabine in advanced pancreatic carcinoma (APC). Methods: In total, 23 patients (pts) with inoperable APC were randomly assigned to gemcitabine combined with two different Atu027 schedules (0.235 mg/kg once weekly vs. 0.235 mg/kg twice weekly). ClinicalTrials.gov Identifier: NCT01808638. Results: The treatment was well-tolerated. There were Grade 3 adverse events (AEs) in 9/11 pts (arm 1) and 11/12 pts (arm 2), while Grade 4 AEs were reported for two pts in each arm. The AEs were mainly laboratory abnormalities without clinical significance. The median progression-free survival reached statistical significance in patients who had metastatic disease (1.6 vs. 2.9 months, *p* = 0.025). Disease control during treatment was achieved in 4/11 pts (arm 1) and in 7/12 pts (arm 2). Pts in arm 1 experienced stable global health status while pts in arm 2 reported improvement. Conclusions: Combining Atu027 with gemcitabine is safe and well tolerated. In pts with metastatic APC, twice-weekly Atu027 is associated with significantly improved outcomes. Our clinical results support the significant involvement of the vascular endothelium in the spread of cancer, and thus the further investigation of its target role.

## 1. Introduction

Pancreatic ductal adenocarcinoma (PDAC) is one of the most aggressive malignancies, and a leading cause of cancer death worldwide. It is expected to become the second leading cause of cancer-related death within this decade [1,2] because, unlike for other solid tumors, there are still no effective therapeutic strategies.

In patients with inoperable disease and better performance status, two chemotherapy options, FOLFIRINOX (fluorouracil, leucovorin, irinotecan, and oxaliplatin) and gemcitabine/nab-paclitaxel, have emerged in the last decade as front-line standards of care, while gemcitabine monotherapy is reserved for patients with lower performance status, substantial comorbidities or other contraindications.

However, in each of these studies, the median overall survival of patients remained less than one year, supporting the ongoing need to develop more beneficial therapies for this disease [3,4].

Patients who showed progression while receiving gemcitabine (+nab-paclitaxel) had a phase III-proven chance of further therapy, with a platinium- or irinotecan-based strategy combined with flouropyrimidine infusion, if the performance status was sufficiently maintained [5,6,7]. After the first line with FOLFIRINOX, a strategy change to gemcitabine/nab-paclitaxel is possible, but is rarely feasible and lacks any phase III-proven overall survival benefit.

The lack of effective targeted agents, as well as missing validated predictive biomarkers that can probably facilitate therapeutic decision-making, are major barriers in the treatment of pancreatic cancer.

Atu027 belongs to a novel class of investigational agents known as RNA interference (RNAi) therapeutics, which are highly targeted pharmacologic inhibitors for silencing specific gene expressions through an enzymatic process that is triggered by small interfering RNA (siRNA) molecules [8]. Atu027 is directed against protein kinase N3 (PKN3) messenger ribonucleic acid (mRNA) expression in the vascular endothelium, and the latter supposed to be involved in metastasis formation. Atu027 comprises four components, encompassing a chemically synthesized double-stranded ribonucleotide (23-mer blunt-ended chemically stabilized siRNA), a cationic lipid (AtuFect01 (β-(L-arginyl)-2,3-L-diaminopropionic acid-N-palmityl-N-oleyl-amide tri-hydrochloride)) and a neutral helper lipid and polyethylene glycol (PEG)-coated lipid, which become lipoplexed in an isotonic sucrose carrier [9].

Data from animal models have shown encouraging results for Atu027 in the reduction/prevention of tumor growth, local invasion and metastases [10,11]. Data from a 28-day (one cycle), first-in-human, phase I dose-escalating clinical trial of single and repeated doses of Atu027 in 34 pts with advanced solid tumors have been published [12,13]. Atu027 was well tolerated (>90% of all adverse events were confined to Grade 1 or 2), safe up to the maximum dose level tested (0.336 mg/kg), and did not raise any serious clinical safety or tolerability concerns. Just under half of the subjects (41%) had stable disease (according to the response evaluation criteria in solid tumors (RECIST)) [14] 1 week after the last Atu027 administration, suggesting the antitumor activity of Atu027.

Here, we report the safety, efficacy and pharmacokinetics (PK) of Atu027 when used in combination with gemcitabine in subjects with locally advanced or metastatic pancreatic adenocarcinoma. All pts were screened for possible prognostic biomarkers. The data have been presented in part, in a poster format, both at ASCO-GI 2016 and at the DKK (German Cancer Congress) 2018.

## 2. Subjects and Methods

### Experimental Design

This was a phase Ib/IIa study of a combination therapy with gemcitabine and Atu027 in subjects with locally advanced or metastatic pancreatic adenocarcinoma (Clinical trials.gov: NCT018086389, EudraCT number: 2012-004429-26) performed in compliance with good clinical practice guidelines and the Declaration of Helsinki, having been approved by the local Ethics Committees. The trial comprised three periods, and patients were enrolled between March 2013 and July 2014.

**Figure 1:**
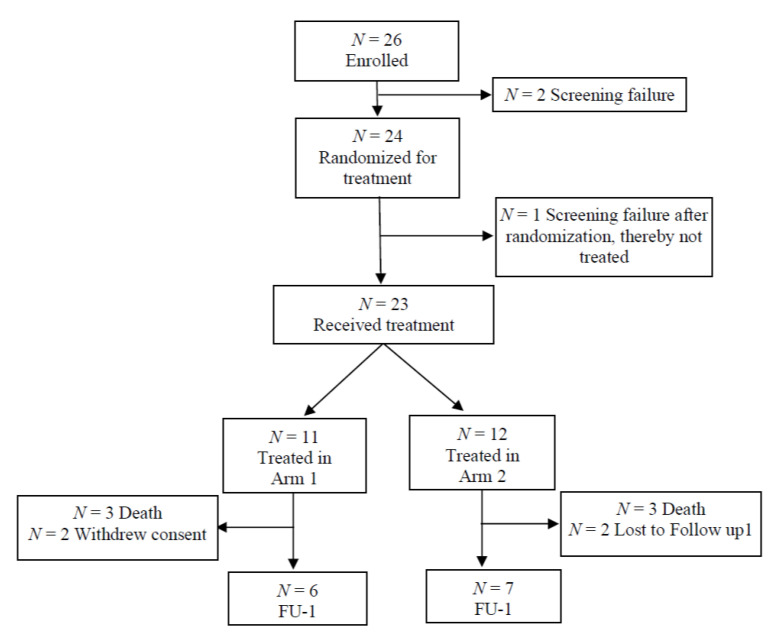
Subject disposition during the treatment period. FU-1=follow-up period 1.

First, there was a safety lead-in period involving 3 subjects with conventional treatment-refractory non-pancreatic cancer who received twice-weekly Atu027 for 4 weeks and once-weekly gemcitabine (one 28-day treatment cycle). Subjects of the safety cohort were included consecutively at 2-week intervals. If one of the subjects in the safety cohort experienced an unacceptable toxicity, the safety cohort was to be expanded to six subjects. If one of these additional three subjects experienced an unacceptable toxicity, the study was to be stopped. After all the subjects finished one 28-day cycle, the safety data were reviewed by an independent data safety monitoring board (DSMB) (Figure 1).

As no safety concerns were observed, subjects with locally advanced or metastatic pancreatic adenocarcinoma were enrolled into the treatment period. This comprised an open-label, parallel-group, 2 arm treatment period that evaluated two dosing schedules of Atu027 and gemcitabine combination therapy.

**Figure 2:**
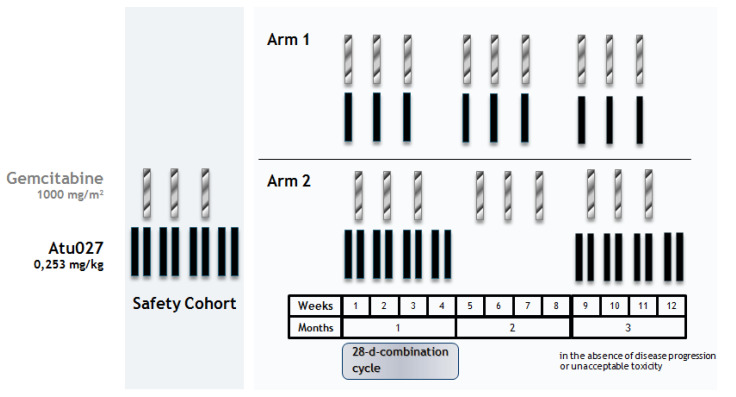
Assessment cycle in Arms 1 and 2.

During this period, subjects were treated in a 28-day cycle (Figure 2), as follows:

- Arm 1 (*n* = 11): Atu027 0.253 mg/kg intravenous (i.v.) and gemcitabine 1000 mg/m^2^ i.v. once weekly for 3 consecutive weeks (on Days 1, 8 and 15) followed by 1 week of no treatment.
- Arm 2 (*n* = 12): Gemcitabine 1000 mg/m^2^ i.v. once weekly for 3 consecutive weeks (on Days 4, 11 and 18) and Atu027 0.253 mg/kg i.v. twice weekly for 4 consecutive weeks (on Days 1, 4, 8, 11, 15, 18, 22 and 25). This 28-day combination cycle was followed by a 28-day gemcitabine-monotherapy cycle.

Gemcitabine was to be given at 1000 mg/m² by 30-minute i.v. infusion, using the Dubois formula with the body surface area evaluated at baseline, to guarantee maximal and optimal pharmacological activity and avoid sub-therapeutic dosing for the subjects’ benefit. Dosage modifications between each cycle or within a cycle were to be applied based upon the actual body weight or the grade of toxicity experienced by the subject

The Atu027 dose was 75% of the highest dose tested to be safe in the previous phase 1 dose-escalation study [12]. The first dose in the first cycle was calculated using baseline body weight, and subsequent doses could be adjusted based on body weight (≥10% change or at the investigator’s discretion). After the completion of the 30-min gemcitabine infusion, Atu027 was administered as a 4 h continuous i.v. infusion. Multiple cycles occurred until unacceptable toxicity or progression of disease were reported.

Finally, all subjects entered into a follow-up phase (first visit at 5 weeks after the last treatment, and then once every 4 weeks up to 1 year) and were treated according to individual needs. Subjects were monitored continuously by the DSMB.

Subjects age 18–84 years with locally advanced or metastatic pancreatic adenocarcinoma stage III/IV, indicated for gemcitabine treatment, were eligible for the trial. No previous chemotherapy with a palliative intention was allowed. The other inclusion criteria included a good clinical performance status (Eastern Cooperative Oncology Group (ECOG) performance status 0–2), an expected life expectancy ≥3 months, an alanine aminotransferase (ALT) or aspartate aminotransferase (AST) ≤3× the upper limit of normal (ULN; ≤5× ULN for subjects with liver metastases), total bilirubin of ≤2.0× ULN (liver metastasis <5 × ULN), serum creatinine of ≤1.5× ULN, adequate bone marrow function (absolute granulocyte count ≥1500 × 10^6^/L and platelet count 100,000 × 10^6^/L) prior to the initiation of a cycle, and a prothrombin time-international normalized ratio/partial thromboplastin time <1.5× ULN. The subjects needed to have at least one measurable lesion according to RECIST Version 1.1.

The main exclusion criteria included the following: pregnancy; hepatitis B or C or HIV infection; those eligible for surgical resection or radiation with curative intent; interstitial pneumonia or extensive and symptomatic interstitial fibrosis of the lung; history of major cardiac disease; poorly controlled diabetes, hypertension or seizure disorder; renal dialysis; and previous or concomitant anticancer chemotherapy, immunotherapy or radiotherapy.

Toxicities were graded according to the National Cancer Institute Common Terminology Criteria for Adverse Events (NCI-CTCAE). Blood and urine aliquots were taken to monitor safety parameters, including hematology and clinical chemistry, during the study.

Tumor size was monitored by abdominal magnetic resonance imaging (MRI) or computed tomography (CT) tumor measurements performed at baseline and on Day 1 of Cycle 3 and on each subsequent odd-numbered cycle in both arms (i.e., once/8 weeks), staged according to RECIST Version 1.1 and reviewed by an experienced local radiologist.

Biomarkers were analyzed with DiscoveryMAP, a quantitative immunoassay service product that measures >300 biomarkers, to identify analytes that change from baseline and biomarkers that predict or monitor the treatment effects of the combined treatment.

The EORTC self-administered 30 item questionnaire Version 3 (comprising five functional scales, three symptom scales, global health, a quality of life scale and 6 single items; 25) was completed at baseline and on Day 1 of all cycles (except Cycle 1) in Arm 1, and on Days 4, 11 and 18 in Cycle 1 and Day 1 on all other odd-numbered cycles in Arm 2.

Blood and urine were collected in the first 4 subjects/treatment arm on Days 1, 8 and 15 of Cycles 1 and 2 and Day 1 of Cycle 3 in Arm 1, and on Days 1, 4, 8, 11, 15 and 18 of Cycle 1 and Day 1 of Cycle 3 in Arm 2, to determine the following PK variables: maximum concentration (C_max_), area under the curve (AUC), time to maximum concentration (t_max_), and the half-life (t_½_) of Atu027 siRNA single strand (A-strand), AtuFect01 and the helper lipid DPyPE.

All subjects enrolled in the treatment period and receiving at least one infusion of Atu027 or gemcitabine were included in the safety and efficacy analyses. All parameters were analyzed descriptively using standard statistical methods.

Data analysis was performed at the end of the treatment period and at the end of the 1-year follow-up period. All data are from baseline of the treatment period up to and including the first follow-up, unless otherwise stated.

A *post-hoc* analysis was conducted in subjects with metastatic pancreatic adenocarcinoma.

## 3. Results

### 3.1. Patient Demographics, Characteristics and Disposition

#### 3.1.1. Lead-in Safety Period

The lead-in safety period of the study (safety cohort) enrolled and treated three subjects with non-pancreatic cancer—two completed the 28-day treatment cycle and one discontinued prematurely due to clinical progression. All three subjects were Caucasian females, 54–69 years of age, with a BMI 24–34 kg/m² and an ECOG baseline performance status of Grade 1 (*n* = 2) or 0 (*n* = 1).

#### 3.1.2. Treatment Period

All subjects were Caucasians (61% female). The treatment arms were comparable for all demographic characteristics, with the exception of age (slightly older in Arm 2 than Arm 1; Table 1). Over two-thirds of the subjects (70%) had an ECOG performance status Grade 1 at baseline, and the remainder an ECOG 0. Of the 24 subjects randomized for treatment, 1 was a screening failure, so 23 subjects received treatment (Arm 1: *n* = 11; Arm 2: *n* = 12) (Figure 1). The mean duration on Atu027 was 2.7 months in both arms, and for gemcitabine 2.7 months (Arm 1) and 2.9 months (Arm 2).

### 3.2. Safety Results

#### 3.2.1. Lead-in Safety Period

In the safety cohort, there were 14 adverse events (AEs), but none of the events were judged to be related to Atu027 or gemcitabine. Out of the 14 AEs, 2 were considered as serious AEs (SAEs) of dysphasia, and peripheral oedema with erysipelas was experienced by one subject. Both recovered without sequelae.

#### 3.2.2. Treatment Period

During the treatment period, AEs were experienced by all but one subject. There were more events in Arm 2 (Table 2). This was primarily due to a higher incidence (difference of greater than or equal to three subjects) in Arm 2 versus Arm 1 of decreased platelet count (67% vs. 27%) and anaemia (50% vs. 27%). Other common AEs are given in Table 2. The *post-hoc* analysis found that the incidence of AEs in subjects with metastatic pancreatic adenocarcinoma was similar in both arms (Arm 1: 100%; Arm 2, 90.0%), but the number of AEs was higher in Arm 2 (*n* = 129) than Arm 1 (*n* = 67).

In total, 49 AEs (experienced by 5 subjects in Arm 1 and 7 subjects in Arm 2) were judged to be related to Atu027, and 110 AEs (experienced by 8 subjects in Arm 1 and 11 subjects in Arm 2) were judged to be related to gemcitabine. The most frequently reported Atu027-related AEs were fatigue (*n* = 5), decreased neutrophil count (*n* = 3), nausea (*n* = 2), vomiting (*n* = 2), peripheral oedema (*n* = 2) and pyrexia (*n* = 2). All other Atu027-related events were reported by one subject each. The most frequently reported gemcitabine-related AEs were decreased platelet count (*n* = 10), fatigue (*n* = 7), anaemia (*n* = 7), decreased neutrophil count (*n* = 6), peripheral oedema (*n* = 6), nausea (*n* = 5) and vomiting (*n* = 5).

Two subjects (both in Arm 2) reported six AEs due to an interaction between Atu027 and gemcitabine (phlebitis, decreased platelet count, hypoalbuminemia, decreased total protein count and vasculitis in one subject, and pyrexia in the second subject).

There were no differences in the severity of AEs between the two arms (Table 2). Four subjects (17%) reported Grade 4 AEs (*n* = 1 increased lipase level, *n* = 1 hyperglycemia, *n* = 2 decreased neutrophil count).

A total of 23 SAEs was reported by 16 subjects. Only two SAEs were judged to be related to treatment—one Grade 3 SAE of pneumonia in Arm 1 was judged to be possibly related to both Atu027 and gemcitabine, and one Grade 2 SAE of fever in Arm 2 was judged to be possibly related to gemcitabine (both recovered without sequelae).

Treatment was suspended in two subjects due to SAEs of pneumonia (*n* = 1) and ileus (*n* = 1), and stopped in one subject due to catheter site extravasation.

Six subjects (three in each arm) died during the treatment period up to and including the first follow-up (*n* = 5 malignant neoplasm progression, *n* = 1 multi-organ failure). None of the deaths were judged to be related to either treatment.

Nine subjects died during the follow-up period (three died due to disease progression and one due to multi-organ failure in Arm 1, and five due to disease progression in Arm 2).

### 3.3. Efficacy Results

#### 3.3.1. Objective Response (OR)

An OR (stabilization, complete response or partial response) was observed in three subjects in Arm 1 and six in Arm 2 at the start of Cycle 3, three subjects in Arm 1 and five in Arm 2 at the start of Cycle 5, one subject in each arm at the start of Cycle 7, one subject in Arm 1 at the start of Cycle 9.

Similar results were also observed in the *post-hoc* analysis of the subgroup with metastatic pancreatic adenocarcinoma.

Tumor size decreased during early cycles (up to Cycle 5), with no noticeable differences between treatment groups. At later cycles, tumor sizes fluctuated.

Across different lesion sites and cycles, a partial response or stable disease at a non-target lesion was observed in 17–100% of subjects in Arm 1, and 10–80% in Arm 2. The liver was the most commonly affected site in all cycles, followed by lymph node and lungs.

Out of the subjects with a study visit at Day 1 of Cycle 3 (i.e., the first time point when occurrence of new lesion was assessed) or later, a new lesion was noted for all subjects in Arm 1, while only half of the subjects in Arm 2 experienced a new lesion at some time during their study participation. Overall, new lesions occurred most commonly in the liver.

#### 3.3.2. Median Progression-Free (mPFS) and Median Overall Survival (mOS)

**Figure 3:**
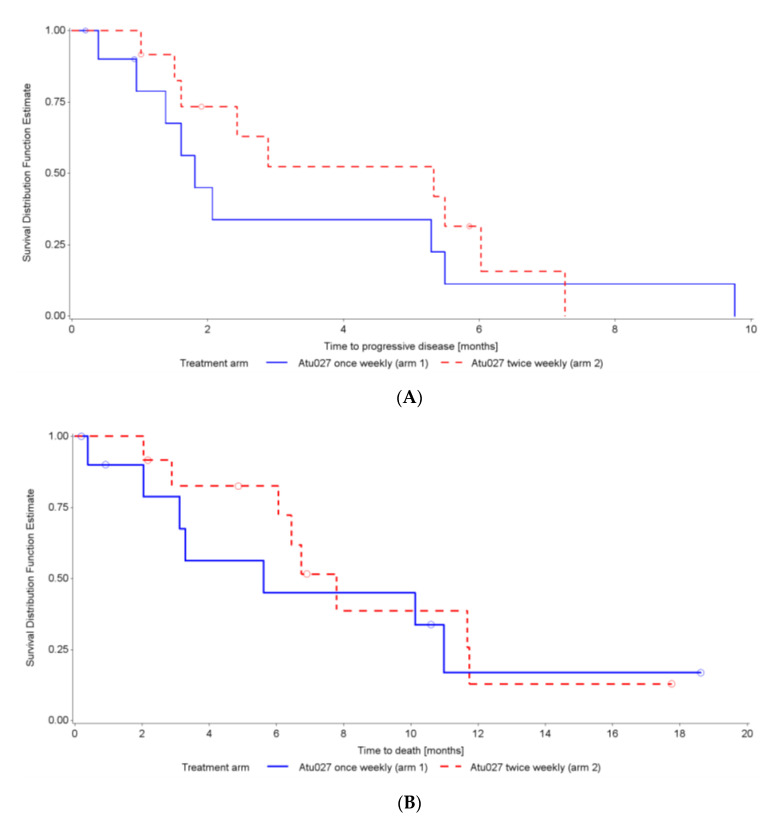
Kaplan–Meier survival curves for progression-free survival (**A**) and overall survival (**B**) (Safety analysis set, *N* = 23).

The median PFS was longer in Arm 2 than in Arm 1, with Kaplan–Meier estimates of 5.33 (95%CI: 1.51–6.02) versus 1.81 (95%CI: 0.39–5.49) months, respectively (*p* = 0.3985; 22% censored; Figure 3A). The *post-hoc* analysis of those with only metastatic disease showed an mPFS of 1.61 (95%CI: 0.39–2.07) months in Arm 1 versus 2.89 (95%CI: 1.02–7.26) months for Arm 2 (*p* = 0.0247; 21% censored).

A similar trend was observed for mOS, with 5.6 months in Arm 1 versus 7.8 months in Arm 2 (*p* = 0.6082; 35% censored; Figure 3B). In the *post-hoc* analysis of those with only metastatic disease, the mOS was 3.3 months in Arm 1 versus 6.7 months in Arm 2 (*p* = 0.5943; 26% censored).

#### 3.3.3. Tumor Markers and Biomarkers

During treatment, the CA 19-9 decreased at most time points during treatment in both arms. CEA (Carcinoembryonic Antigen) was unchanged or reduced in Arm 1, but increased in Arm 2.

Of the 301 analytes assessed as potential biomarkers, 46 and 59 analytes changed significantly from baseline in Arms 1 and 2, respectively, and 13 biomarkers were significantly associated with response status. Once adjusted using the Benjamini–Hochberg method for multiple hypothesis testing, only one analyte (Factor VII in Arm 1) changed significantly from baseline, and one (Angiopoietin-2) was significantly associated with response status at the first follow-up.

#### 3.3.4. ECOG Performance Status

There was little change in performance status across all time points in both treatment arms. More subjects in Arm 2 had performance status improvements (i.e., change of –1) than in Arm 1 at most time points.

#### 3.3.5. EORTC Quality of Life (QoL) Questionnaire

**Figure 4:**
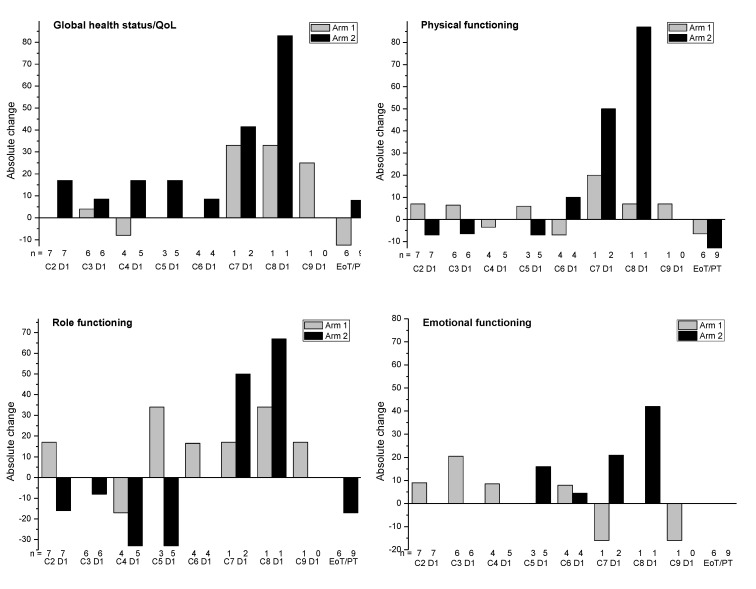

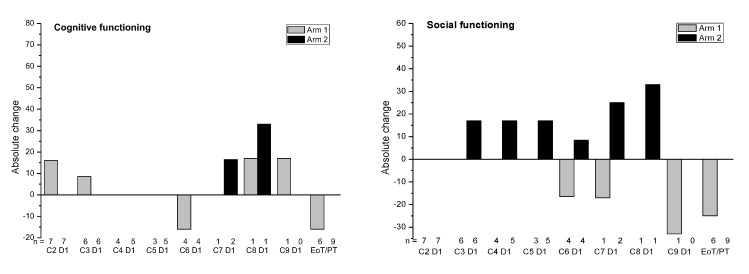
Absolute change from baseline in the global health status/quality of life and functional scales (EORTC) during the treatment period (Safety analysis set, *N* = 23). C = cycle; D = day; EoT = end of treatment; FU-1 = follow-up visit 1; PT = premature termination.

The QoL and symptom scales improved or were unchanged at most time points in both arms, and the functional scores deteriorated or improved at different timepoints in both arms (Figure 4). Overall, improvements appeared to be more commonly observed in Arm 2.

### 3.4. Pharmacokinetics

A total of 11 subjects was included in the PK analysis—the 3 subjects from the lead-in safety period, 4 from Arm 1 and 4 from Arm 2. PK analysis found that Atu027 siRNA increased gradually during the 4-h infusion, then decreased quickly after 4–6 h, with a slower terminal elimination phase from 6 h onwards. AtuFect01 and the helper lipid, DPyPE, also increased gradually during the infusion, but plateaued between 4 and 8 h, with a slower terminal elimination phase from 8 h onwards.

T_max_ for all compounds was achieved at or just after the end of infusion (4 h). The mean t_1/2_ was ~10 h for Atu027 siRNA, ~20 h for AtuFect01 and ~17 h for DPyPE. The C_max_ and AUC of Atu027 siRNA remained relatively constant over the consecutive administrations with both once- (Arm 1) and twice-weekly (Arm 2) administration (~150 ng/mL for C_max_ and ~1000 h·ng/mL for AUC). However, there were differences between Arms 1 and 2 for AtuFect01 and DPyPE—C_max_ and AUC remained relatively constant over the consecutive administrations of once-weekly Atu027 (AtuFect01 and DPyPE, respectively: ~6500 and ~5500 ng/mL for C_max_ and ~110,000 and ~93,000 h·ng/mL for AUC), but tended to increase over the consecutive administrations of twice-weekly Atu027 administration (AtuFect01 and DPyPE, respectively: from C_max_ of 6775 and 5522 ng/mL at Cycle 1, Day 1, to 9991 and 7675 ng/mL at Cycle 1, Day 18, and from an AUC of 123,375 and 98,685 h·ng/mL at Cycle 1, Day 1, to 182,571 and 136,133 h·ng/mL at Cycle 1, Day 18). There was no evidence of an effect of concomitant gemcitabine treatment on the PK of any of the three analytes.

## 4. Discussion

This phase I/II study investigated the safety, efficacy and PK of Atu027 when given in combination with gemcitabine. In addition, the patients were screened intensively for prognostic or predictive biomarkers. There were two arms in this trial. In Arm 1, gemcitabine and Atu027 were administered once weekly for 3 weeks, followed by a 1-week rest. In Arm 2, Atu027 was administered twice weekly and gemcitabine once weekly for 3 weeks, followed by a 1-week rest, in odd-numbered cycles, and gemcitabine monotherapy once weekly for 3 weeks, followed by a 1-week rest, in even-numbered cycles. Subjects were treated until unacceptable toxicity or disease progression were seen.

There was a good safety profile in both treatment arms, with only two treatment-related SAEs—one event of pneumonia (Grade 3) judged as related to both Atu027 and gemcitabine in Arm 1 and one of fever (Grade 2) related to gemcitabine in Arm 2. Both SAEs recovered without sequelae. There were more AEs per subject in the twice-weekly versus the once-weekly Atu027 arm, but there were no differences between arms in the severity of the AEs (as judged by NCI-CTCAE grading). This trend was also seen in the *post-hoc* analysis of subjects with metastatic pancreatic adenocarcinoma. Twice as many AEs were judged to be related to gemcitabine (*n* = 110) as to Atu027 (*n* = 49). Consistent with the phase 1 study 22, the most frequent Atu027-related AE was fatigue; however, this was never dose-limiting. Other common Atu027-related AEs included decreased neutrophil count, nausea, vomiting, peripheral oedema and pyrexia.

Overall, twice-weekly Atu027 showed greater clinical benefits than once-weekly administration. There was a trend of a better OR (stabilization, complete response or partial response) in Arm 2 than in Arm 1 (e.g., Cycle 3 = 60% vs. 50%; Cycle 5 = 100% vs. 50%; first follow-up: 71% vs. 33%). In addition, the subjects treated with twice-weekly Atu027 showed longer mPFS and mOS, better ECOG performance status and fewer new lesions than those given once-weekly Atu027. There were no differences between the two arms in tumor markers or tumor size. The differences between Arm 2 and Arm 1 in the improvement of OR, mPFS and mOS were even more pronounced in the *post-hoc* analysis of subjects with metastatic pancreatic cancer. This study was not focused on survival data, but at least Arm 2 looks promising when comparing it to the historical data with a median OS for gemcitabine of 3.3 months [3].

Reduced levels of the tumor markers CEA and CA 19-9 are associated with improved OS in patients with pancreatic adenocarcinoma [15]. In our investigation, CA 19-9 decreased at most time points during treatment in both arms, but there were variable effects on CEA.

The PK data are consistent with those reported previously in the first-in-human study [12]. The t_max_ was achieved at approximately 4 h (end of the infusion) for the Atu027 siRNA single strand (A-strand), AtuFect01 and the helper lipid, DPyPE. The mean t_1/2_ was ~10 h for Atu027 siRNA, ~20 h for AtuFect01 and ~17 h for DPyPE. There was no accumulation in plasma of any of the three compounds following once-weekly Atu027 administration. However, twice-weekly Atu027 administration resulted in an approximately 40–50% plasma accumulation of AtuFet01 and DPyPE on Day 18 versus Day 1 of Cycle 1. As no side effects were observed that could be specifically due to either AtuFect01 or DPyPE, the clinical relevance of this is unclear, but further investigation is warranted, particularly in view of the potential superior efficacy of twice-weekly Atu027. Possibly, different pharmacokinetics might account for the superiority of Arm 2.

There was no evidence of an effect of concomitant gemcitabine treatment on the PK of any of the three analytes. However, we did not analyze in detail the prognostic markers influencing tumor sensitivity to Gemcitabine, such as, e.g., ENT1, Notch3 or MiR-21.

A phase I/IIa study of another siRNA drug (*siG12D-LODER™*, a biodegradable implant that encompasses anti-KRASG12D siRNA) in combination with weekly gemcitabine-based chemotherapy or modified FOLFIRINOX in subjects with locally advanced pancreatic cancer [16] reported a similar safety and efficacy profile to that reported here with Atu027. At least three other siRNA agents are under investigation in subjects with inoperable solid tumors (e.g., TKM-080301, APN401, DCR-MYC and Eph-A2).

There are some limitations to this phase I/II study. The number of subjects included in each arm is small. It should also be noted that the mean t_1/2_ data must be considered with caution in view of the short time interval over which they were estimated, and/or the large between-subject variability.

Nowadays, more intensive treatment regimens are used as the first-line strategy in fitter patients. With the observed low drug interaction and clinically insignificant toxicity of ATU 27 in combination with gemcitabine, the substance can be further investigated with more intensive regimens.

In addition to that, it can even be given to people with comorbidities, making it an ideal candidate for the first combination with a targeted compound, as has been used here. At present, supportive data are lacking on whether adding targeted therapeutics to intensive regimens, such as FOLFIRINOX or Gemcitabine/nab-paclitaxel, offers any added value. Recently, the pegylated human hyaluronidase (PEGH20) combined with gemcitabine and nab-paclitaxel failed to improve median overall survival (HALO-301 trial, NCT02715804) [17].

Pancreatic cancer is not only resistant to many standard cancer strategies—it is also not very responsive to targeted or immunotherapy approaches, as has been exemplarily demonstrated by a phase Ib trial using Pembrolizumab [18]. Atu027 has a different approach, as it is not supposed to work as a cytotoxic drug itself, but as a compound targeting the endothelium via PKN3, therefore delaying or preventing metastases, most likely through an enhanced barrier function or antiangiogenic mechanism. Therefore, further trials should focus on combination or sequential treatment strategies, e.g., with gemcitabine/nab-paclitaxel. Data pertaining to its action in an adjuvant setting are nonexistent so far, but due to the bad prognosis, even in this context a clinical trial with Atu027/gemcitabine should give further insight into its efficacy.

In conclusion, the combination therapy of Atu027, a targeted agent that induces RNAi as well as the down-regulation of the PKN3 mRNA transcript and protein directly in the vascular endothelium, with gemcitabine had a good overall safety and tolerability profile. Atu027 administered once weekly had a slightly better safety profile than Atu027 twice-weekly. Moreover, the PK analysis revealed no accumulation of any compounds in plasma following once-weekly Atu027 administration, while there was an accumulation of the AtuFect01 and DPyPE lipids with twice-weekly Atu027 administration. The significance of this is unclear.

Subjects given twice-weekly Atu027 had better clinical responses, in terms of OR, mPFS, mOS and ECOG performance, than those given once-weekly Atu027. We would therefore suggest regimen two for further combination trials, such as with gemcitabine/nab-paclitaxel, ideally in a randomized way.

## 5. Conclusions

Pancreatic cancer is amongst the most deadly cancers worldwide, and continues to respond poorly to current standard chemotherapies as well as targeted and immunotherapies. Therefore, innovative treatment approaches are required.

Atu027 is an RNA interference (RNAi) therapeutic specifically silencing PKN3 mRNA expression in the vascular endothelium, thereby potentially inhibiting the metastatic expansion of the tumor. The findings of this clinical investigation imply the efficacy benefit in patients with metastatic tumors when treated with this compound. Further trials combining Atu027 with more intensive regimens are warranted. If confirmed, this study could serve as a proof-of-principle for a novel class of agents inhibiting the metastatic process rather than targeting tumor growth.

## Figures and Tables

**Table 1:** Baseline characteristics of subjects randomized to the treatment period (Safety analysis set, *N* = 23).

Baseline Characteristics	Arm 1 *N* = 11	Arm 2 *N* = 12
Sex		
Male: n (%)	4 (36.4)	5 (41.7)
Female: n (%)	7 (63.6)	7 (58.3)
Race		
Caucasian: n (%)	11 (100)	12 (100)
Median age: years (range)	64 (43–74)	71 (44–80)
BMI: kg/m^2^ (standard deviation)	24.4 (3.9)	23.3 (2.5)
ECOG: n (%)		
0	4 (36.4)	3 (25.0)
1	7 (63.6)	9 (75.0)
Pancreatic adenocarcinoma		
Type		
Locally advanced: n (%)	2 (18.2)	2 (16.7)
Metastatic: n (%)	9 (81.8)	10 (83.3)

BMI = body mass index, ECOG = Eastern Cooperative Oncology Group.

**Table 2:** Adverse events during the treatment period (Safety analysis set, *N* = 23).

Adverse Events	Arm 1 (*N* = 11)	Arm 2 (*N* = 12)
Subjects with AEs, n (%)	11 (100.0)	11 (91.7)
AEs, n	116	147
Anaemia, n (%)	3 (27.3)	6 (50.0)
Nausea, n (%)	5 (45.5)	7 (58.3)
Vomiting, n (%)	3 (27.3)	5 (41.7)
Peripheral oedema, n (%)	3 (27.3)	5 (41.7)
Fatigue, n (%)	5 (45.5)	5 (41.7)
Pyrexia, n (%)	2 (18.2)	3 (25.0)
Neutrophil count decreased, n (%)	2 (18.2)	3 (25.0)
Platelet count decreased, n (%)	3 (27.3)	4 (33.3)
Grade, n (%)		
1	9 (81.8)	11 (91.7)
2	10 (90.9)	11 (91.7)
3	9 (81.8)	11 (91.7)
4	2 (18.2)	2 (16.7)
5	3 (27.3)	2 (16.7)
Subjects with SAEs, n (%)	8 (72.7)	7 (58.3)
Grade, n (%)		
2	0 (0.0)	3 (27.3)
3	8 (72.7)	4 (36.4)
SAEs, related to Atu027, n	1	1

Likely, probably, possibly, or definitely related to treatment; AE = adverse event, SAE = serious adverse event.
